# Infants expect agents to minimize the collective cost of collaborative actions

**DOI:** 10.1038/s41598-022-21452-5

**Published:** 2022-10-12

**Authors:** Olivier Mascaro, Gergely Csibra

**Affiliations:** 1 Université Paris Cité, CNRS, Integrative Neuroscience and Cognition Center, F-75006 Paris, France; 2 Cognitive Development Center, Department of Cognitive Science, Central European University, Vienna, Austria; 3 Department of Psychological Sciences, University of London, Birkbeck, UK

**Keywords:** Psychology, Human behaviour

## Abstract

This paper argues that human infants address the challenges of optimizing, recognizing, and interpreting collaborative behaviors by assessing their collective efficiency. This hypothesis was tested by using a looking-time study. Fourteen-month-olds (*N* = 32) were familiarized with agents performing a collaborative action in computer animations. During the test phase, the looking times were measured while the agents acted with various efficiency parameters. In the critical condition, the agents’ actions were individually efficient, but their combination was either collectively efficient or inefficient. Infants looked longer at test events that violated expectations of collective efficiency (*p* = .006, *d* = 0.79). Thus, preverbal infants apply expectations of collective efficiency to actions involving multiple agents.

## Introduction

Humans are unique in the extent to which they willfully collaborate, i.e., act together to achieve shared goals. Essentially, if humans were unable to act collectively efficiently, collaboration would be of little benefit^1^. Moreover, since collaboration is so central to human life, being able to predict and interpret the unfolding of others’ collaborative behavior is crucial. In this paper, we describe one cognitive mechanism that allows humans to plan, predict, and interpret collaborative actions: the capacity to compute collective efficiency. Previous studies have shown that from infancy onwards, humans expect agents to be efficient, i.e., to minimize the costs of their individual actions^2–8^. Here we report a study that investigated whether infants’ expectation of efficiency generalizes to collective actions. Throughout this paper, we assume that a collaborative action is collectively efficient if it achieves its intended effect while minimizing the aggregate costs to each of the individual collaborators.

Computations of collective efficiency are likely to play a central role in the interpretation of collaboration. One can infer that two people are collaborating not only when they have committed, verbally, or non-verbally, to achieve a goal together^9,10^, but also from attending to their actions. For instance, when watching firefighters forming a human chain, whereby individuals would pass buckets to each other to extinguish a fire, the relations between their actions reveal that they are aiming at achieving a shared goal together^9,11–13^.

Conventional wisdom holds that collaborative behaviors can be identified by recognizing fixed spatio-temporal relations between individual actions, such as synchronicity, contingent reactivity, or similarity. However, these cues are not always present, nor they are sufficient for the interpretation of collaborative actions. For example, similarity between two individuals’ behaviors is rarely an appropriate cue for identifying instances of collaboration, and in many cases, collaborative actions require that partners act in markedly different ways^14,15^. Furthermore, two competing (e.g., fighting) individuals may act in synchrony and react to each other, even though they are certainly not collaborating^11^. And, perhaps most importantly, using fixed spatial–temporal cues to detect instances of collaboration does not allow one to identify shared goals. Even if an observer detects, using simple spatio-temporal cues, that two people are collaborating, she would still not know what shared goal the individuals are trying to achieve, or how they might attempt to achieve it.

We propose that the recognition and interpretation of collaborative actions can be achieved by assessing collective efficiency. According to this view, the representation of collaborative actions builds on mechanisms that are recruited to interpret individual actions. The prediction and interpretation of individual actions can be achieved by assuming that agents are rational — i.e., they aim to maximize the benefits while minimizing the costs of their actions^2–4,6–8^. These expectations of rationality guide humans’ representation of individual actions and goals from infancy onwards^2,4,5,8,16,17^.

Rationality expectations can also be used to assess collaborative actions. The recognition and interpretation of collaborative behaviors can be achieved by assuming that collaborators tend to be collectively efficient, i.e., to minimize collective costs invested to achieve a certain goal. Collective costs can be computed by aggregating the costs to each of the individual collaborators. This notion of collective efficiency applies to all kinds of collaborations, including sequential ones, where the collaborators do not act simultaneously (for instance, when A and B collaborate to displace objects, such that A first passes objects to B, before B places the objects in their final location).

Indeed, collaborative actions are often collectively efficient, such that each individual action reduces the collective costs of collaboration^18,19^. For example, adults seem to take into account the costs of their partners’ actions when holding the door for someone^20^, or when passing an object for someone to place it in a specific location^21–24^. People also transfer objects in a collectively efficient manner by selecting paths that minimize the aggregate costs of movement for the dyad^25,26^.

Unlike relying on spatio-temporal cues, the assumption of collective efficiency of collaborative actions can be used to evaluate hypotheses about shared goals by inverting computations that plan collectively efficient actions^27^. The expectation of collective efficiency can also support action predictions (by assuming that given a set of constraints, agents aim to achieve their shared goal in a collectively efficient manner). In short, we propose that by applying the expectation of efficiency at the collective level, it is possible to recognize, predict, and interpret collaborative actions.

In this study, we tested whether infants expect agents to minimize the collective costs of collaborations. This project builds on infants’ capacity to identify the goals of individual actions^6,28–33^ and collaborations^10,34–38^. To clarify: our aim was not to assess whether infants can encode shared goals—this question has already been addressed successfully by multiple studies^10,34–38^. Rather, we assessed whether infants apply expectations of efficiency to collaborative interactions between multiple agents.

We used a violation-of-expectation paradigm, a method capitalizing on infants’ tendency to look longer at events that they find unexpected or hard to process^39,40^. Our study used self-propelled geometrical shapes to represent agents —just like in^2,28–30,32^. Simplified stimuli depart from infants’ daily experiences, yet, they have key benefits. Naturalistic stimuli contain extraneous information that may divert infants’ attention or generate experimental confounds. By contrast, if properly built, simplified stimuli can tap precisely into the specific cognitive mechanisms that they are designed to test^41^. Whether the fruitful exploitation of simplified stimuli in infant research is due to the fact that they generate illusions in infants (i.e., infants think that they watch real agents acting in front of them) or to the fact that they are interpreted as representations of fictional events on a screen^42^ is a further concern, which, however, is orthogonal to the question addressed by this study.

**Figure 1:**
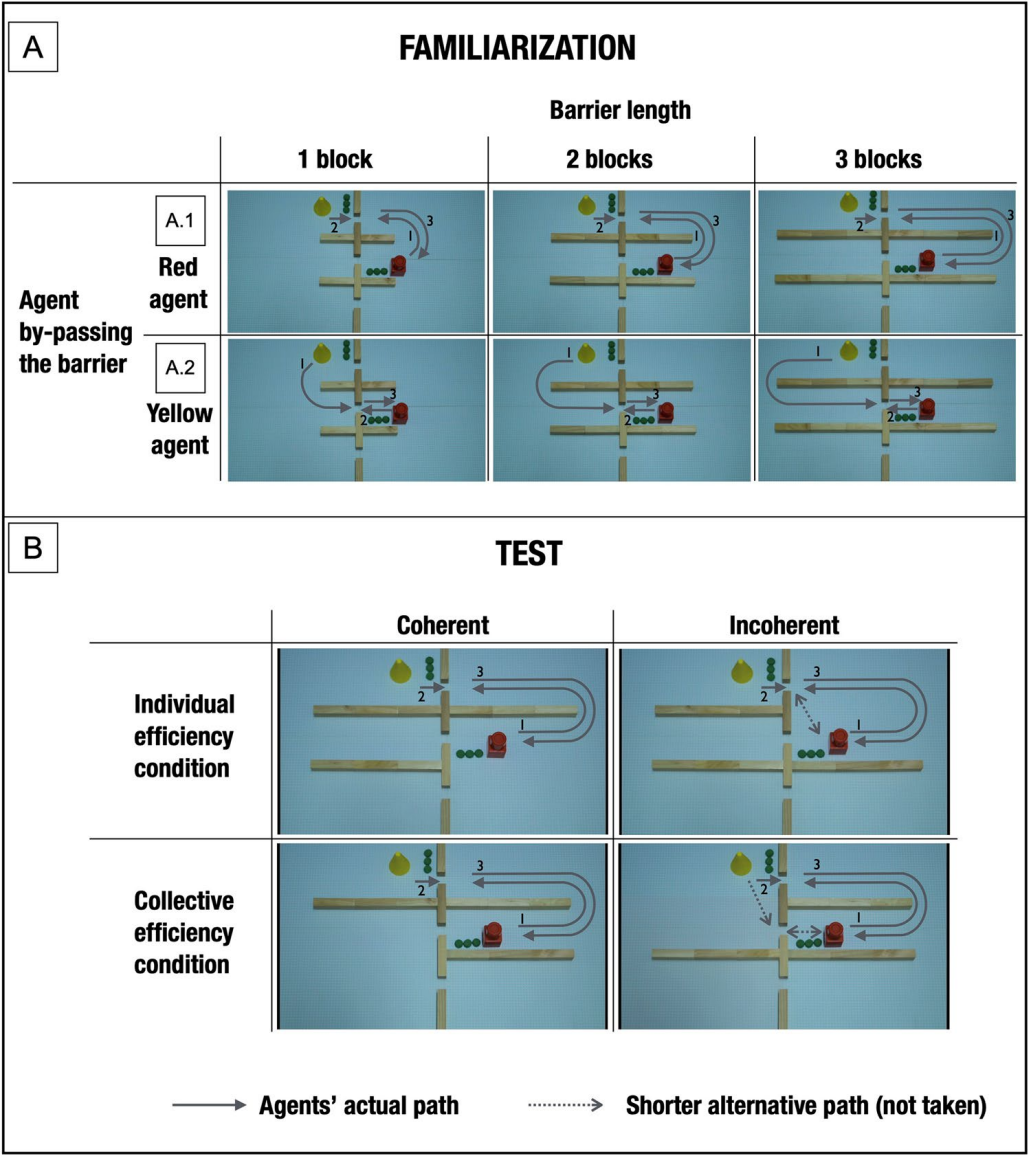
Schematic illustration of familiarization (**A**) and test (**B**) movies. The grey solid arrows represent the path taken by agents in the videos. The numbers (in black) indicate the order of actions. The dotted line arrows represent available alternative shorter paths that were not taken by the agents. The arrows are used for illustrative purposes only — they did not appear in the movies shown to the participants.

During familiarization, the participants observed two agents performing a sequential collective action. The agents transferred a ball from one location to another (videos S1, S2). This collaboration was composed of two individual actions. First, an agent collected a ball and pushed it through a gap in a wall. Next, a second agent picked up the ball and pushed it towards its final location (Fig. 1A). Thus, our stimuli depicted actions that have analogs outside the lab. Many instances of collaboration encountered in daily life involve two agents displacing objects from one location to another—such as, for instance, people forming a human chain, or passing objects to each other. To perform their actions, the agents in our stimuli had to bypass one of four barriers whose length varied across familiarization movies (from 1 to 3 identical blocks). Each of the two agents bypassed the barrier in half of the familiarization movies (Fig. 1 A.1–2).

**Figure 2:**
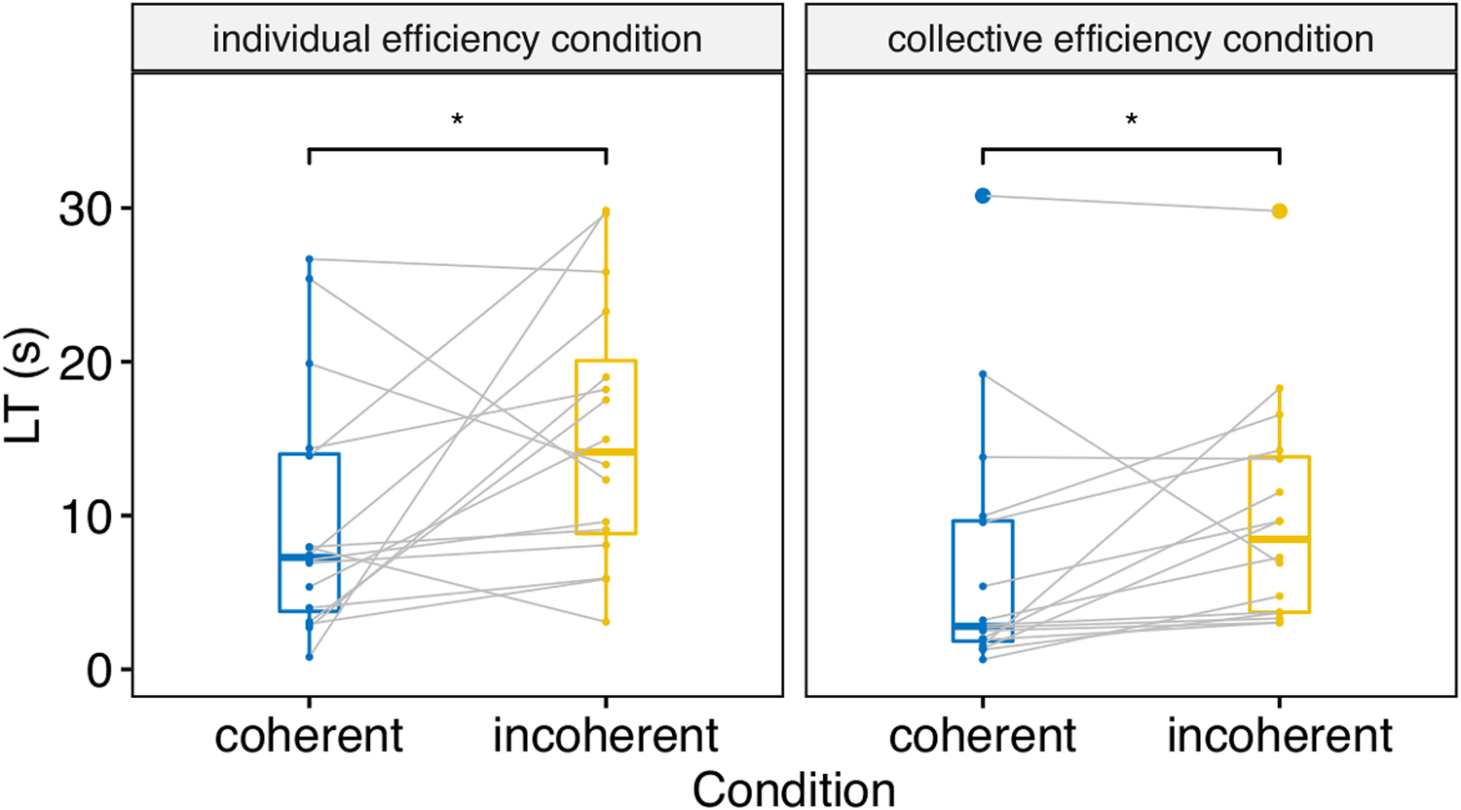
Boxplot of untransformed looking times to test events as a function of Condition and Test coherence (Coherent vs. Incoherent). Dots represent individual data points; grey lines connect repeated measures from individuals. *p < .05 by Wilcoxon signed-rank test.

During the test, infants saw the agents achieve the same goal while acting in an efficient manner (coherent test event), or in an inefficient manner (incoherent test events). We manipulated across conditions whether expectations of efficiency were violated at the individual or collective level. To manipulate the relative efficiency of actions, we operationalized individual costs as the path length travelled by each individual agent^2,4^. Accordingly, we operationalized collective costs as the sum of the path length travelled by all individuals participating in a collaborative action.

In the incoherent test of the individual efficiency condition, one of the agents made an unnecessary detour to reach the gap through which the ball was transferred (Fig. 1B, first row, second column, video S3). In the coherent test of the individual efficiency condition, the agent’s detour was necessary to bypass a barrier and reach the gap through which the ball was transferred (Fig. 1B, first row, first column, video S4). In the incoherent test of the collective efficiency condition, the agents’ individual actions were efficient with respect to their own subgoals, when the two agents’ actions were considered separately (Fig. 1B, second row, second column, video S5). One of the agents made a detour that was necessary to bypass a barrier and reach the gap through which the ball was transferred. The other agent used the shortest available route to transfer the ball. However, the combination of these individually efficient actions was suboptimal with respect to the overarching goal because there was an alternative route available with a shorter total path length. In the coherent test of the collective efficiency condition, there was no available alternative that would make the total path length of the agents shorter (Fig. 1B, second row, first column, video S6). At the end of each test movie, the image froze from the moment the agents had completed their actions. We measured the looking time from this time point until the infant looked away for 2 s or more, or after 30 s had elapsed, at which point the test trial ended.

The individual efficiency condition served to validate our stimuli and data analysis procedure. Previous studies have shown that infants look longer when their expectations of individual efficiency are violated, for instance when agents take an unnecessarily long path rather than the shortest available route to achieve their goal^2–4,4,5,7^. Thus, we hypothesized that in the individual efficiency condition, infants would look longer at the incoherent than at the coherent test. Similarly, for the collective efficiency condition, we assumed that if infants detect violations of collective efficiency, they would look longer at the incoherent test than at the coherent test.

We tested 14-month-olds because, by this age, infants’ capacity to identify joint goals and complex individual actions composed of several steps is well-established^36,43–45^. In a complementary study, we also tested younger infants’ capacity to assess the collective efficiency of collaborative actions, but the results were inconclusive (see the Supplementary Materials).

## Results

A first analysis confirmed that condition (individual vs. collective efficiency) had no effect on looking times at familiarization videos (*M* = 61.31; *SD* = 4.15 vs. *M* = 60.09; *SD* = 5.82, t (30) = 0.74, *p* = 0.465; independent sample t-test). This first analysis was performed at the request of an anonymous reviewer. The further analyses reported below focused on looking at test events, and they were all planned.

We ran a mixed-model ANOVA on looking times at test events with Test coherence (coherent vs. incoherent test) as a within-subject factor, and with Order of test trials (coherent vs. incoherent test first) and Condition (individual efficiency vs. collective efficiency) as between-subject factors. This ANOVA revealed a main effect of Condition (*F *(1, 24.13) = 4.78, *p* = 0.039), indicating that the looking times at test events were longer in the individual efficiency condition than in the collective efficiency condition. The ANOVA also revealed a main effect of Test coherence (*F *(1, 23.68) = 18.11, *p* < 0.001), indicating that 14-month-olds looked longer at incoherent test events than at coherent test events. Moreover, we found a two-way interaction between Order of test trial and Test coherence (*F *(1, 23.68) = 8.49, *p* = 0.008), showing that 14-month-olds’ tendency to look longer at incoherent test trials was stronger when incoherent test trials were presented first. This interaction was due to the fact that looking times tended to be longer in the first than in the second test trial — an effect not unprecedented in infancy research^2^.

Planned comparisons confirmed that infants’ looking times were longer for incoherent test events than for coherent test events both in the individual and collective efficiency conditions (see Fig. 2 and Table 1).

## Discussion

We have found that infants assess the collective efficiency of collaborations and use this competence to form expectations about the way agents perform collaborative actions. In our study, infants discriminated between coherent and incoherent test events in the collective efficiency condition. They did so *not* by expecting one of the individual agents to minimize its own individual efforts or the efforts of the other agent. Rather, to succeed in the task, infants needed to perform computations that took into account the path lengths of both agents. Had infants considered the agents’ actions separately, they would not have produced the looking patterns we obtained because the two agents performed identical, and individually efficient, actions in the coherent and incoherent tests of the collective efficiency condition (see Fig. 1B). Only if they considered the first agent’s action in relation to the action that the second agent was forced to perform as its consequence could infants realize that this sequence of actions was suboptimal in the given situation.

Several explanations may account for the 14-month-olds’ expectations of collective efficiency. In our view, the most plausible explanation is that infants assess the efficiency of a collective action by processing it as a complex action composed of sub-parts that are achieved by physically distinct effectors (Note, though, that adults’ collaborative actions tend to be also collectively efficient when they cannot be divided into components this way^46^). This view is consistent with theories of team reasoning^47–50^, and shared-effort models^20^, and postulates that infants perform efficiency computation on the aggregate of the costs of the two agents’ actions. Our data do not tell whether infants’ sensitivity to the collective efficiency of collaborations result from more complex forms of recursive reasoning involving representations of each agent’s inferences about their partners’ strategies, costs, and benefits^51–55^. Thus, the level of strategic reasoning that infants would attribute to agents engaging in collaborative actions, and whether infants assume that agents consider the efforts of their partners at all await future research. Similarly, our data do not clarify whether infants would expect agents to act in a collectively efficient manner even in the absence of previous evidence for collaboration, or whether they would cease to show this expectation when the two agents appear to be competing instead of collaborating. Thus, future research could investigate the factors that trigger or limit infants’ expectations of collective efficiency.

Nevertheless, our data suggest that 14-month-old infants apply expectations of efficiency over actions involving several distinct agents, thereby showing that assumptions of collective efficiency develop early. We suspect that infants use overlapping—if not identical — mechanisms to assess the efficiency of collaborations involving several distinct agents and to assess the efficiency of complex individual actions composed of subparts. In other words, our results suggest that humans can process collaborative behaviors by extending their capacity to reason about the efficiency of individual actions to sets of agents.

It should be noted that in our framework, expectations of rationality are used to assess goal hypotheses, not to generate them. Thus, our data do not say much about how infants form the hypothesis that agents might collaborate to achieve a shared goal. However, our data suggest that once infants generate a hypothesis about agents collaborating to achieve a specific shared goal, they can use collective efficiency computations to form expectations about agents’ actions. By comparing these expectations to the agents’ actual behaviors, infants can confirm or deny the hypothesis that agents are collaborating to achieve the hypothesized shared goal.

It has been argued that infants could infer affiliation relationships by assessing whether agents incorporate a social partner's utility into their own utility function^56^. Our results provide indirect support for this hypothesis by showing that, by their second year of life, infants can determine whether collaborating agents take into account their partners’ costs. Whether, in addition to this, infants expect agents who minimize the collective costs of their collective actions to be affiliated with each other is an important question for future research.

Our movies in the collective efficiency condition presented actions that were always efficient individually while their combination was optimal or sub-optimal at the collective level. Thus, our results highlight that assessments of efficiency are always relative to a specific frame of reference within which rationality is expected to apply^47^. By showing that human infants can evaluate the efficiency of collaborative actions, our results open many novel questions about how humans combine the costs and benefits of multiple agents, how infants (and adults) determine the frame of reference within which rationality is expected to apply, and how they compute the respective contributions of agents engaging in collaborative actions.

## Method

### Participants

Data collection took place between May and December 2013. Two groups of 16 14-month-old infants participated (individual efficiency condition: *M*_*age*_ = 441 days, range = 426–455 days; collective efficiency condition: *M*_*age*_ = 443 days, range = 431–450 days). Details about our recruitment procedure, the way we set sample sizes, and exclusion criteria are reported in the supplementary materials.

### Setup

Infants were tested in a dimly lit soundproof room. They were seated on their caregiver’s lap 100 cm from a 40-inch LCD monitor on which the stimuli were presented. A hidden camera (temporal resolution: 25 frames/s) recorded the infants’ looking behavior. The caregivers were instructed to close their eyes during the entire procedure.

### Procedure

Infants were presented with movies generated by stop-motion animations showing agents represented by self-propelled geometrical figures. The agents engaged in a collaborative action (transferring small balls from one location to another). First, infants were presented with six familiarization trials to familiarize them with the agents’ collaboration. We used a fixed number of familiarization trials, drawing on past studies of infants’ processing of goal-directed actions^17,28,30,57^. The familiarization phase was followed by two test trials. During each familiarization and test trial, the participants were shown a single movie while a soft tune was played in the background. The trials were interspersed with a looming stimulus on a black background to attract attention to the screen.

### Familiarization trials

Familiarization movies were the same under all conditions. In each of them, two self-propelled agents (3D geometrical shapes) collaborated to transfer a small ball from one location to another. First, an agent (a yellow cone) collected the small ball, transported it to a gap between wooden blocks, and pushed it through the gap. On the other side of the gap, another agent (a red cylinder attached to a cubic base) took the ball and placed it on a stack of balls. To perform their actions, the agents had to bypass one of four barriers whose length varied across familiarization movies (Fig. 1A) These barriers could measure one, two, or three identical wooden blocks, and the duration of the familiarization movies varied accordingly (one block: 9 s, two blocks: 11 s, three blocks: 13 s). For each barrier length, the agent by-passing the barrier was the yellow agent once (Fig. 1 A.1), and the red agent once (Fig. 1 A.2). In each familiarization movie, the agents transferred only one ball. At the end of each familiarization movie, the agents froze for a fixed duration of 3 s. The factors that were counterbalanced during the familiarization trials are reported in the Supplementary Materials.

### Test trials

After the familiarization phase, infants were presented consecutively with a coherent and an incoherent test movie (order of presentation counterbalanced across participants). The test movies were identical to the familiarization movies in which all barriers had a length of three blocks, but we changed the constraints on the agents’ actions by editing out one of the four barriers from the scene (see Fig. 1B). In all conditions, for each participant, the agents followed the same path in the two test movies; thus, coherent and incoherent test trials differed not in the action they depicted but in the environments in which those actions were performed. We counterbalanced across participants whether, during the test, agents transferred the ball using the gap that was closest to the yellow agent’s initial position, or the gap that was closest to the red agent’s initial position.

### Ethical approval

The study was approved by an independent ethical review committee (the Hungarian Ethical Review Committee for Research in Psychology; EPKEB, code: 2013/9), and the parents of all participants gave their written informed consent before inclusion. All experiments were performed in accordance with the ethical rules and standards regarding psychological experimentation in Hungary.

**Table. 1:** Means and standard deviations of looking times to test events and their statistical comparisons across conditions.

Looking times (s)			Statistical comparisons					
	Coherent	In Coherent	Paired t-test			Wilcoxon test		
	*M (SD)*	*M (SD)*	t(15)	*p*	d [95%CI]	*W* +	*p*	R_rb_ [95%CI]
Individual efficiency condition	9.78 (8.05)	15.35	− 2.23	.041	− 0.56	29	.044	− .57 [− .84, − .09]
Collective efficiency condition	6.80 (8.30)	9.94 (7.31)	− 3.17	.006	-0.79 [− 1.35, − .22]	21	.013	− .69 [− .88,− .28]

## Coding and data analysis

We coded the video recordings frame-by-frame to determine whether infants looked at the screen or looked away. Details about our coding procedure and inter-rater agreement are reported in the Supplementary Materials. Prior to analysis, the looking-time data were log-transformed^58^. We performed parametric analyses on transformed data, and, when appropriate, equivalent non-parametric analyses were performed on untransformed data. For ease of reading, we report only the means and standard deviations of the untransformed data along with these analyses. Transformed and untransformed data are available in the Supplementary Materials. Due to violations of assumptions of homoscedasticity, we used the Welch-James approximate degrees of freedom (ADF) to evaluate the significance of F values when running ANOVAs^59,60^. These analyses were conducted in R using the package ‘welchADF’^61^. All statistics reported in this paper are two-tailed.

## Supplementary Information

Supplementary Information 1.

Supplementary Legends.

Supplementary Video 1.

Supplementary Video 2.

Supplementary Video 3.

Supplementary Video 4.

Supplementary Video 5.

Supplementary Video 6.

## Data Availability

Raw and log-transformed data can be found in the Supplemental Materials.
