# Supplementary material for: Infants expect agents to minimize the collective cost of collaborative actions
Source: Sci Rep. 2022 Oct 12;12:17088. doi: 10.1038/s41598-022-21452-5 (PMC9556639; doi:10.1038/s41598-022-21452-5)
Supplement: Supplementary file 1 — Supplementary Information 1. [file 41598_2022_21452_MOESM1_ESM.pdf]

Infants Expect Agents to Minimize the Collective Cost of Collaborative Actions

(Supplementary Materials)

Olivier Mascaro

Université Paris Cité, INCC UMR 8002, CNRS, F-75006 Paris, France

Gergely Csibra

Cognitive Development Center, Department of Cognitive Science, Central European University, Vienna, Austria

Department of Psychological Sciences, Birkbeck, University of London, UK

Supplementary Materials

Supplementary Methods For the Main Study

Participants

Participants were recruited by sending letters to a randomly selected sample of children born in the Budapest area. Our sample sizes (n = 16 per condition) were set after comparable looking time studies testing 14-month-old infants' understanding of collaborative actions, which reported positive results with an effect size equal to 0.76[1]. Assuming comparable effect sizes in our studies, a sample size of 16 was sufficient to reach a power equal to .81 for evaluating the effect of Condition on looking times by two-tailed paired t-tests with $\alpha = .05$ (analysis performed with G*Power (v.3.1))[2].

In addition to the 32 fourteen-month-old participants retained in the analysis, thirteen additional infants participated but were excluded from analyses because of inattentiveness, i.e., looking for less than 75% of the duration of familiarization movies or not looking at the outcome of the agents 'actions during the test (5), crying or unwillingness to complete the experiment (5), parental interference (2), and impossibility to code the participants' gaze behaviors (1).

Procedure: Counterbalanced Factors

Familiarization trials were presented in one of two orders counterbalanced across participants: 1Y-3R-2R-2Y-1R-3Y or 1R-3Y-2Y-2R-1Y-3R, where numbers stand for the length of the barrier (1, 2 or 3 blocks), Y stands for transferring the ball through the gap closest to the yellow agent's initial position, and R stands for transferring the ball through the gap closest to the red agent's initial position. We also counterbalanced across participants whether the initial position of the balls and of the agents was on top or at the bottom of the screen, or on the right or left side of the vertical wall. The movements of the agents varied

accordingly.

Coding procedure

We coded frame-by-frame from the video recordings whether infants looked at the screen or looked away. Blinks were considered as looks away if they lasted for more than 0.2 s. Infants looking for less than 75% of the duration of familiarization movies or not looking at all at the outcome of the event in either test trial were considered inattentive and were excluded from analysis.

The data were coded by the first author, and 50% of the data was also randomly selected and recoded by a second coder unaware of the hypothesis of the study. The correlations between the coders' measures of looking times were high for each combination of condition (individual vs. joint) and test coherence (coherent vs. incoherent) (average r = .99, range = .96-1.00). When the difference between the values from the first and the second coder exceeded 20% of the first coder's value, the discrepancy was resolved by discussion.

Supplementary Study

Background

In a complementary study, we also tested 9-month-old infants' capacity to assess the collective efficiency of collaborative actions. We wanted to determine whether below a certain age, children might track the efficiency of individual actions, without assessing yet the collective efficiency of collaborative actions. Nine-month-olds appeared to be an appropriate age group to test this possibility. When they observe agents acting, 10-month-olds appear to have difficulties processing novel actions composed of several steps — unless they receive additional information by performing the action themselves, or by observing the overarching goal of the action being achieved directly[4–6]; see[7] for a counterexample. These data make it plausible that 9-month-olds might react to violations of individual efficiency, but not

necessarily to violations of collective efficiency, which require them to fuse two sequential actions as means towards a single outcome.

Method

Participants

Two groups of 16 9-month-old infants participated (individual efficiency condition: M_{age} = 268 days, *range* = 250-292 days; individual efficiency condition: M_{age} = 270 days, *range* = 258-288 days). The recruitment procedure was the same as for the study reported in the main text. Twenty-one additional infants participated but were excluded from analyses because of inattentiveness, i.e., looking for less than 75% of the duration of familiarization movies or not looking at the outcome of the agents 'actions during the test (11), crying or unwillingness to complete the experiment (4), parental interference (1), technical failure (2), and experimental error (3).

Apparatus, Procedure, Coding and Data Analysis

The apparatus, procedure, coding and data analysis pipeline were identical to those

used in the Study reported in the main text.

Results

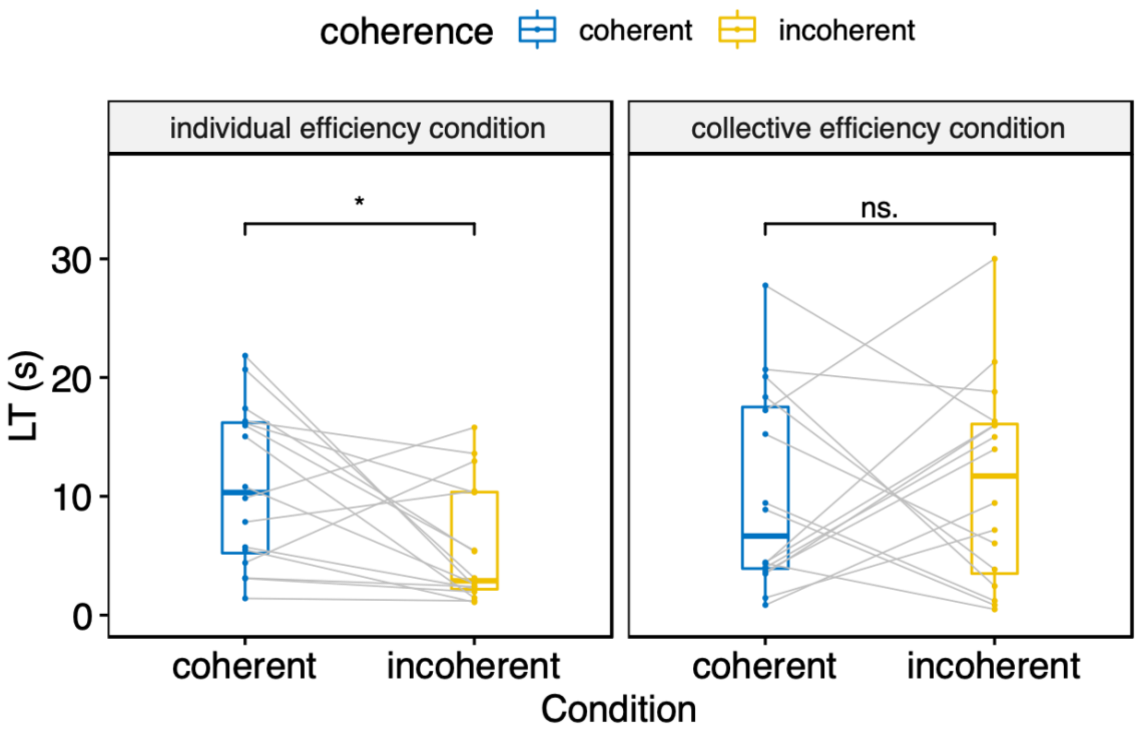

Figure S1.

Box-plot of untransformed looking times to test events in the Supplementary Study as a function of Condition, and Test coherence (Coherent vs. Incoherent). Dots represent individual data points; grey lines connect repeated measures from individuals. * $p < .05$ by Wilcoxon signed-rank test

Two analyses were performed on looking times at familiarization videos at the request of an anonymous reviewer. The first of these analyses confirmed that condition (individual vs. joint) had no effect on nine-month-old infants' looking times at familiarization videos (M = 61.61; SD = 3.92 vs. M = 61.86; SD = 4.54, p = .91; two-sample t-test). In a second analysis requested by an anonymous reviewer, we also compared looking at familiarization across age groups —pooling data from joint and individual conditions. We ran an ANOVA on looking time at familiarization movies, with Age (9- vs. 14-month-old) and Condition (individual vs. collective efficiency) as between-subject factors, This analysis revealed no significant effect,

in particular no effect of Age (9- vs. 14-month-old) on infants' looking times at familiarization videos (F(1, 60) = .87, p = .354), and no interaction between Age and Condition (F(1, 60) = .42, p = .521). The analyses reported below focused on looking at test events, and they were all planned.

The ANOVA ran on the 9-month-olds' data revealed a main effect of Test coherence (F(1, 25.92) = 5.48, p = .027), indicating that the average looking times at test events were longer for coherent than for incoherent test events. The ANOVA also revealed a two-way interaction between Condition and Test coherence (F(1, 25.92) = 5.09, p = .033), suggesting that 9-month-olds' tendency to look longer at coherent test trials was stronger in the individual efficiency condition. Moreover, we found a two-way interaction between Order of test trial and Test coherence (F(1, 25.92) = 15.58, p < .001) and a three-way interaction between Order of test trial, Test coherence, and Condition (F(1, 25.92) = 18.77, p < .001). We have no definitive account for these interactions with Order of test trial. Importantly, they bear no consequences for the interpretation of our results.

Planned comparisons revealed that in the individual efficiency condition 9-month-olds' average looking times were significantly larger for coherent test events (10.94 s, *SD* = 6.72 s) than for incoherent test events (5.77 s, *SD* = 5.06 s; see Figure S1, and Table 1). In contrast, in the collective efficiency condition, there was no significant effect of Test coherence on looking times (see Table S1).

Table S1

Means and standard deviations of looking times to test events and statistical comparisons across conditions for the Supplementary Study

	Looking Times (s)		Statistical Comparisons					
	Coherent	Incoherent	Paired t-test			Wilcoxon test		
	M (*SD*)	*M* (*SD*)	$t(15)$	p	d [95%CI]	$W+$	p	r_{rb} [95%CI]
9-month-olds								
Individual Efficiency Condition	10.94 (6.72)	5.77 (5.06)	3.13	.007	.78 [.21, 1.34]	112	.021	.65 [.21, .87]
Collective Efficiency Condition	10.27 (8.37)	11.17 (8.59)	.034	.973	.01 [-.48, .50]	59	.669	-.13 [-.60, .40]

Discussion

In our Supplementary Study, 9-month-olds reacted to violations of individual efficiency only, but not to violations of collective efficiency. This result dovetails with studies showing that, before 10 months of age, infants often find it hard to infer the overarching goal of novel actions composed of several steps[4–6]. Importantly, the effect of coherence on 9-month-olds' looking time in the individual efficiency condition was due to longer looking at coherent than at incoherent test movies (i.e., the opposite of what we anticipated). In light of infants' well-established tendency to look longer at events in which agents act inefficiently rather than efficiently[8], this is a surprising finding. We do not have a definitive explanation for this reverse effect, which calls for caution when interpreting the results of our Supplementary Study.

References

1. Henderson, A. M. & Woodward, A. L. "Let's work together": What do infants understand about collaborative goals? *Cognition* **121**, 12–21 (2011).
2. Faul, F., Erdfelder, E., Lang, A.-G. & Buchner, A. G* Power 3: A flexible statistical power analysis program for the social, behavioral, and biomedical sciences. *Behav. Res. Methods* **39**, 175–191 (2007).

3. Csibra, G., Hernik, M., Mascaro, O., Tatone, D. & Lengyel, M. Statistical treatment of looking-time data. *Dev. Psychol.* **52**, 521 (2016).
4. Henderson, A. M., Wang, Y., Matz, L. E. & Woodward, A. L. Active experience shapes 10-month-old infants' understanding of collaborative goals. *Infancy* **18**, 10–39 (2013).
5. Sommerville, J. A., Hildebrand, E. A. & Crane, C. C. Experience matters: the impact of doing versus watching on infants' subsequent perception of tool-use events. *Dev. Psychol.* **44**, 1249 (2008).
6. Sommerville, J. A. & Woodward, A. L. Pulling out the intentional structure of action: the relation between action processing and action production in infancy. *Cognition* **95**, 1–30 (2005).
7. Begus, K., Curioni, A., Knoblich, G. & Gergely, G. Infants understand collaboration: Neural evidence for 9-month-olds' attribution of shared goals to coordinated joint actions. *Soc. Neurosci.* **15**, 655–667 (2020).
8. Gergely, G. & Csibra, G. Teleological reasoning in infancy: The naıve theory of rational action. *Trends Cogn. Sci.* **7**, 287–292 (2003).
