# Supplementary material for: Infants expect agents to minimize the collective cost of collaborative actions
Source: Sci Rep. 2022 Oct 12;12:17088. doi: 10.1038/s41598-022-21452-5 (PMC9556639; doi:10.1038/s41598-022-21452-5)
Supplement: Supplementary file 2 — Supplementary Legends. [file 41598_2022_21452_MOESM2_ESM.pdf]

Supplementary Videos Titles and Legends

Video S1. Example of familiarization video showing the agents collaborating, with the red agent by-passing the barrier.

Video S2. Example of familiarization video showing the agents collaborating, with the yellow agent by-passing the barrier.

Video S3. Example of incoherent test video from the individual efficiency condition.

Video S4. Example of coherent test video from the individual efficiency condition.

Video S5. Example of incoherent test video from the collective efficiency condition.

Video S6. Example of coherent test video from the collective efficiency condition.
